# From plug-and-play to institution-calibrated radiology AI: a practical framework for operationalizing local validation, monitoring and governance

**DOI:** 10.3389/fradi.2026.1902217

**Published:** 2026-07-16

**Authors:** Muhammad Awais, Abdul Rehman

**Affiliations:** 1 Department of Radiology, Aga Khan University Hospital, Karachi, Sindh, Pakistan; 2 Department of Medicine, TidalHealth Peninsula Regional, Salisbury, MD, United States

**Keywords:** algorithm performance monitoring, domain shift, local validation, model governance, radiology artificial intelligence

## Abstract

Radiology artificial intelligence (AI) is increasingly developed on large external datasets and deployed across institutions, but real-world model performance may vary substantially after implementation. Imaging AI interacts with a local ecosystem shaped by scanner hardware, acquisition protocols, reconstruction methods, technologist practices, disease prevalence, patient demographics, reporting conventions, and clinical workflow. These factors can produce domain shift, degrade calibration, alter false-positive and false-negative patterns, and affect clinical utility. In this article, we argue that radiology AI should move beyond a “plug-and-play” deployment paradigm toward institution-calibrated AI stewardship. We propose an institution-specific radiology AI performance profile, conceptually analogous to a local antibiogram, to summarize how AI tools perform within a specific clinical environment. Unlike prior MLOps and radiology AI governance frameworks, the proposed profile translates lifecycle management into a radiology-specific, locally maintainable artifact that captures technical context, clinical context, performance metrics, reference standards, equity checks, workflow effects, and governance triggers. The framework applies not only to diagnostic decision support, but also to research cohort generation and radiology education, where local reporting language, imaging protocols, and case mix may strongly influence model reliability. We emphasize that institution-specific calibration does not require every hospital to develop AI models *de novo*. Rather, externally developed, vendor-based, and foundation-model approaches should be paired with local validation, cautious threshold adjustment, calibration when applicable, surveillance for drift and protocol changes, and multidisciplinary governance. Responsible radiology AI deployment should therefore ask not only whether a model is accurate, but whether it remains accurate, relevant, equitable, and useful locally over time.

## Introduction

The clinical performance of radiology artificial intelligence (AI) is commonly reported as if it were a stable property of the model, but real-world performance depends on the interaction between the model and the local imaging ecosystem in which it is deployed (1). Yet radiology practice is not portable in the same way that software is portable. A chest radiograph, head CT, prostate MRI, or mammogram is the product of a local technical and clinical ecosystem shaped by scanner hardware, detector technology, reconstruction software, MRI field strength, pulse sequence, slice thickness, contrast timing, technologist practice, patient population, disease prevalence, and institutional protocol. Consequently, the clinical performance of an AI model should not be viewed as an intrinsic property of the algorithm alone, but as a property of the interaction between the algorithm and the institution in which it is deployed (2). The relevant implementation question is therefore not whether an imaging AI model is accurate in general, but whether it remains accurate, calibrated, equitable, and clinically useful within the local environment in which it is deployed and used.

A useful analogy may be found in infectious diseases. Clinicians do not assume that antimicrobial susceptibility patterns from one hospital can be transplanted uncritically into another. Instead, institutional antibiograms summarize local organism-specific susceptibility patterns and help clinicians tailor empiric therapy to the microbial ecology of their own health system. Radiology AI requires a similar conceptual shift. The relevant question is not simply whether an algorithm performed well in a published study or at another institution. The question is whether it performs reliably here: on these scanners, with these protocols, for these patients, interpreted by these radiologists, and embedded in this workflow.

The need for local monitoring and institutional calibration after deployment of a machine learning (ML) model has been recognized in the published literature as well as in consensus statements from radiological societies (1–11). Medical imaging AI is vulnerable to domain shift in general, and radiology in particular, where hardware differences, acquisition protocols, reconstruction kernels, scanner upgrades, technologist technique, annotation practices, and patient demographics can degrade model performance (3, 4). A ML model trained on one institution's data may learn features that are clinically meaningful, but it may also learn hidden institutional signatures that can be problematic when deployed in other institutions (4).

To address the issue of commercial ML model deployment in disparate clinical environments, multi-society statements have emphasized the principles of lifecycle management, continuous monitoring, governance, model updating, and implementation science (9, 10). Additionally, MLHOps, MedMLOps, CyclOps, and resilience-aware MLOps have been built as generic toolkits to facilitate deployment of ML models in clinical environments in general (5–8). Our Opinion article adds to this prior work by translating these established principles into a concrete institutional radiology AI profile that is tailored to the clinical workflow of radiologists and radiology departments (Supplementary Table S1 provides a summary of prior works vis-à-vis our proposed framework).

## Institution-specific radiology AI performance profiling

We propose an institutional radiology AI performance profile, conceptually analogous to a local antibiogram: a structured, local, and dynamic summary of model performance metrics across clinical tasks, scanners, protocols, patient subgroups, reference standards, and workflow settings (see Supplementary Figure S1). This would not be a loose dashboard of aggregate model accuracy. Rather, it would be a structured, local, dynamic dashboard describing how each AI model performs across intended use cases, imaging tasks, modalities, scanners, protocols, patient subgroups, comparator or ground truth, operating threshold, alert burden, and workflow settings (a prototype is provided in Supplementary Table S2). For a diagnostic model, core metrics would include sensitivity, specificity, positive predictive value (PPV), negative predictive value (NPV), area under the receiver operating characteristic curve (AUROC), area under the precision-recall curve (AUPRC), false-positive burden, false-negative patterns, alert volume, radiologist concordance, turnaround-time effects, and subgroup performance. These values would ideally be stratified not only by disease label, but also by scanner, protocol, acquisition settings, clinical indication, care setting, and clinically important subgroups.

The institutional radiology AI performance profile is not novel as a philosophical or strategic concept, since these theoretical constructs are already well-described in the published literature (5–11). Rather, its proposed value is operational: it converts an abstract AI stewardship construct into a local, radiology-specific artifact that can be reviewed by radiologists and other stakeholders alike during diverse tasks such as procurement, acceptance testing, quality improvement, and revalidation after scanner, software, protocol, or population changes. A detailed discussion of potential consumers and utility of the institutional radiology AI profile is provided in Supplementary Table S3.

## Clinical, research, and educational use cases

There are several use cases for this institutional radiology AI performance profile. Diagnostic support is the most obvious use case. Consider AI tools for intracranial hemorrhage triage, pulmonary embolism detection, fracture identification, lung nodule characterization, breast density assessment, mammographic cancer detection, or prostate MRI lesion detection. In each case, local disease prevalence and local tolerance for false positives shape clinical utility. A highly sensitive emergency department triage model may be useful if it accelerates care for critical findings, but harmful if it produces excessive alerts, interrupts workflow, or shifts radiologist attention away from unflagged studies. Similarly, a model with its threshold adjusted to one population with unique local disease patterns may become poorly calibrated in another. Institutions should therefore avoid treating a vendor-provided operating point as universally optimal. Local validation, threshold adjustment, and workflow-specific adaptations may be as important as the model architecture itself (see subheading “Local validation, threshold adjustment, and monitoring” for further details).

Beyond diagnosis, institutional performance profiling may monitor and facilitate research cohort generation and education. Since imaging-defined cohorts are difficult to identify using ICD and CPT codes alone, report-based NLP (natural language processing) tools are often utilized. These tools are sensitive to local templates, impression style, and uncertainty language. By monitoring performance of AI NLP tools through precision, recall, and false-positive patterns, institutional performance profiling could demonstrate if such tools are reliable enough for local research cohort generation. Additionally, by focusing on report-language failure modes, this profile may facilitate and guide local adaptations to these tools to make them more reliable. With respect to teaching and education, institutional radiology AI performance profiling can convert local AI/NLP model failures (discordant cases based on protocol-specific pitfalls, subtle findings, etc.) into a monitored teaching-file pipeline, thereby facilitating education. For example, an institution-adapted NLP tool could be used to identify oncology patients with incidental pulmonary embolism on staging or surveillance CT reports. The performance profile would document the tool's local positive predictive value, recall, false-positive causes, and need for manual chart or imaging adjudication before the cohort is used for research. For education, AI-enabled retrieval could identify locally relevant cases of missed fractures on prior radiographs or CT examinations for resident teaching, with attention to local protocol variation, image quality, and common interpretive pitfalls. In both examples, the profile does not replace expert review; rather, it determines whether the retrieval tool is sufficiently reliable to support research screening or teaching-file curation.

## Local validation, threshold adjustment, and monitoring

An institution-calibrated approach does not require every radiology department to build models from scratch. Instead, institutional radiology AI should adopt a hybrid model: broadly trained algorithms, foundation models, and vendor tools should be coupled with local validation, threshold adjustment when needed, local calibration when supported and legally permissible, and continuous post-deployment monitoring. Emerging radiology AI infrastructure provides a practical foundation for this lifecycle approach. ACR AI-LAB enables local evaluation and development of AI models using institutional data (12); Assess-AI supports real-world algorithm performance monitoring (13); and the ACR Recognized Center for Healthcare-AI (ARCH-AI) provides a quality assurance framework for acquisition, deployment, maintenance, use, and monitoring of clinical imaging AI (14). Similar initiatives and proposals have been put forth by other radiological societies and governing bodies including the WHO, NICE, RSNA, RANZCR, ESR and MICCAI (9–11, 15–17). These programs illustrate how professional societies can operationalize local evaluation, governance, and post-deployment monitoring. The key recognition is that the AI lifecycle does not end at installation. It begins there.

A practical local AI stewardship framework would include four steps. First, institutions should perform pre-deployment validation on representative local data, including scanner- and protocol-level subgroup analyses where feasible. These analyses should consider sensitivity, specificity, PPV, NPV, AUROC, AUPRC, false-positive burden, and false-negative patterns. Second, when performance is inadequate, institutions should consider local threshold adjustment, calibration of configurable model parameters (when supported by vendors), and operational modifications to mitigate model shortcomings and support local workflows. Such operational modifications may entail an additional decision support layer, limits on model applicability, and rules governing decisions based on model outputs. Third, once deployed, AI models should be enrolled in continuous monitoring for data drift, software version changes, scanner upgrades, protocol changes, discordance with radiologist interpretation, and unexpected failure modes. Fourth, oversight should be multidisciplinary, involving radiologists, medical physicists, informaticists, data scientists, quality and safety leaders, compliance experts, and frontline users. Each step of this process can be facilitated by institutional radiology AI profiling.

Even institutions without advanced data science infrastructure can begin with a pragmatic minimum standard. Before deployment, radiology departments can assemble a representative local test set, define the intended clinical use case, compare AI output with radiologist interpretation, or accepted reference standards, and examine performance across major scanners, protocols, and care settings. After deployment, departments can track alert volume, radiologist override rates, discordant cases, scanner or protocol changes, and unexpected failure modes. This approach does not require each institution to become an AI development center; it requires each institution to become an informed AI steward.

## Importance of ground truth

Ground truth can be a practical bottleneck for institutional AI calibration and unreliable ground truth labels can undermine institutional AI model performance. For pre-deployment validation, institutions may use enriched local test sets with expert adjudication, pathology, follow-up imaging, clinical outcomes, or tumor board decisions where applicable. For post-deployment surveillance, full adjudication of every case is unrealistic. Instead, a pragmatic approach may be to sample discordant cases, radiologist overrides, false-negative safety events, morbidity and mortality conference cases, peer-review discrepancies, and clinically consequential alerts. NLP tools evaluating follow-up reports may support surveillance, but should not be treated as infallible reference standards. Thus the reliability of the institutional radiology AI profile depends heavily on the reliability of the reference standard.

## Regulatory, governance, resource and cost considerations

The regulatory and legal implications of local calibration are extremely important to keep in mind. The US Food and Drug Administration's predetermined change control plan framework (18) recognizes that AI-enabled devices may require planned modifications over time, while emphasizing validation, implementation methodology, and continued safety and effectiveness. These planned modifications follow pre-specified vendor specifications, which were already embedded in the approved device's labeling. Local adaptation of AI-enabled software must not become uncontrolled modification of medical devices. Although regulatory pathways differ internationally, the underlying distinction remains important: local validation is expected, whereas local modification or retraining will almost certainly carry regulatory implications. Radiology departments should therefore distinguish between local validation, which should be expected; threshold adjustment or operational optimizations, which would be permissible depending on the exact model specifications and institutional governance structure; and model retraining, fine-tuning or modification, which will almost always require vendor involvement and regulatory review. A detailed discussion of these levels of local adjustments and calibration is provided in Supplementary Table S4. Therefore, a radiology AI “antibiogram” should support governance, not bypass it.

Institutional AI governance should also clearly identify who owns the model locally, who reviews performance, who can pause or decommission it, and how liability is shared among the institution, vendor, and clinical users. Resource needs will vary by hospital size and AI maturity, but a practical starting structure includes a radiologist champion, informatics or IT representative, medical physicist, quality and safety lead, and vendor contact (see Supplementary Table S5 for a detailed breakdown of this). Larger programs may require dedicated data science or informatics time. Costs should be judged against potential benefits, including safer deployment, reduced alert fatigue, earlier detection of drift, and avoidance of clinically consequential failures. Additionally, institutional radiology AI profiling should not operate in silos or create a parallel bureaucracy; instead, it should integrate with existing quality improvement (QI) frameworks, such as peer review data (RADPEER scores), morbidity and mortality conferences, lexicon compliance, and structured reporting adherence.

## Discussion

The analogy to antibiograms has limitations. Microbial susceptibility is measured using standardized laboratory methods, whereas AI performance depends on clinical labels (ground truth) that may be imperfect, delayed, subjective, or inconsistently recorded. Imaging AI also affects workflow, radiologist behavior, and downstream testing in ways that are harder to capture in standardized metrics.

Local performance profiling also has equity implications. Aggregate accuracy may conceal systematic underperformance in subgroups defined by age, sex, race, ethnicity, insurance status, body habitus, comorbidity, scanner access, or care setting. A locally monitored AI system should therefore be evaluated not only for mean performance, but also for clinically meaningful performance heterogeneity (see Supplementary Table S6 for a sample checklist).

**Figure 1:**
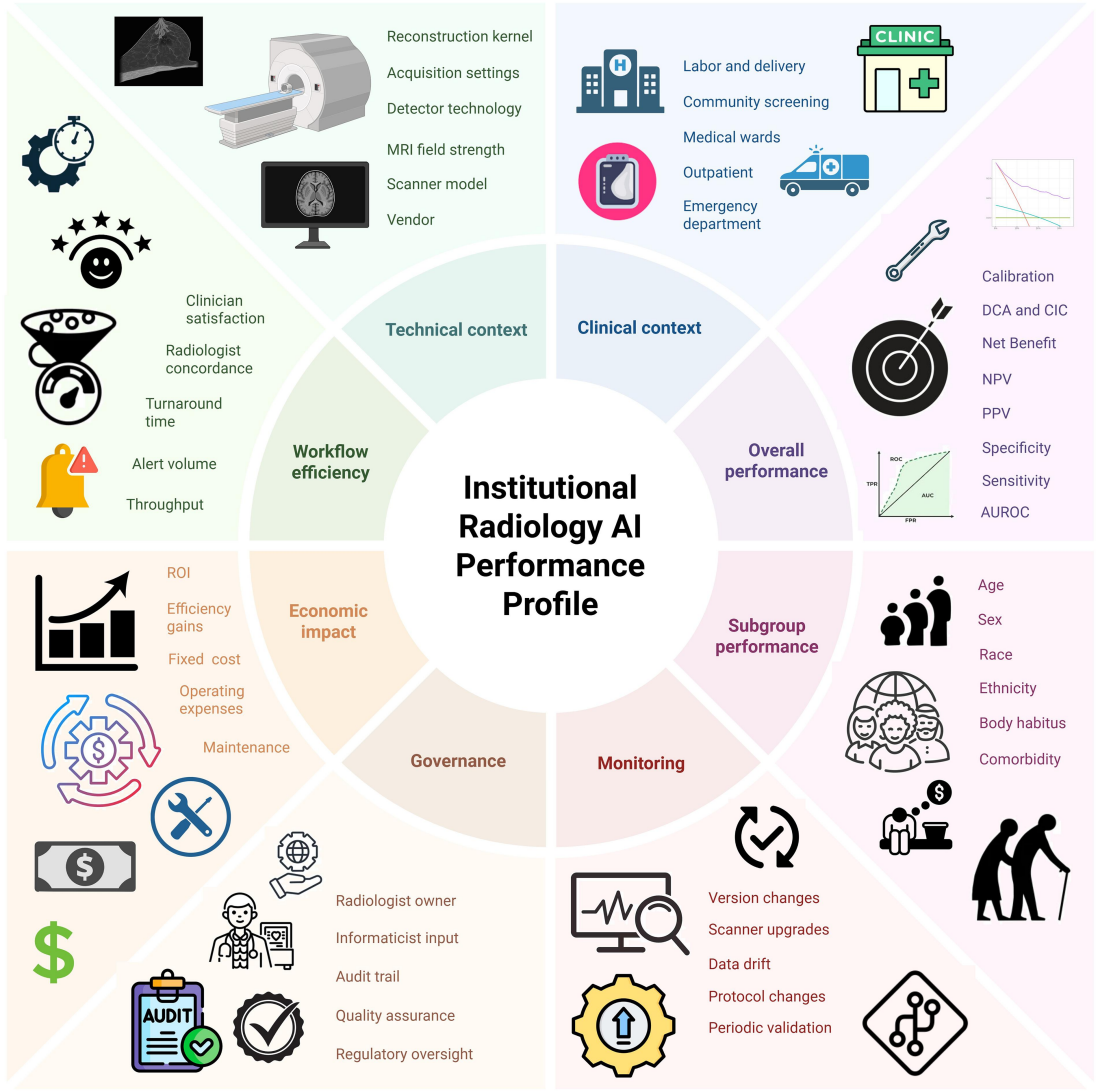
A framework for evaluating key aspects of the proposed institutional radiology AI performance profile (analogous to “antibiogram”). A framework for evaluation of local radiology AI across technical context, clinical context, overall and subgroup performance, monitoring, governance, economic impact, and workflow efficiency. This institution-specific profile supports validation, calibration, deployment, and surveillance of AI models within the local imaging ecosystem. Figure created using BioRender (https://BioRender.com/47s61ra).

Radiology AI should therefore move beyond the plug-and-play paradigm. The future is not AI isolationism, in which each hospital independently develops every tool. Nor is it blind transplantation, in which algorithms are installed with minimal local scrutiny. The future is institution-calibrated AI: externally developed when appropriate, locally validated before use, locally calibrated when permissible and necessary, and continuously monitored after deployment (see Figure 1). Radiology departments should therefore ask not only, “Is this AI model accurate?” but also, “Is it accurate for us, in this setting, over time?” Like an antibiogram, the answer should be local, empirical, and periodically updated; unlike an antibiogram, it should also account for workflow, calibration, subgroup performance, and model drift.
