# Supplementary figures and images for: From plug-and-play to institution-calibrated radiology AI: a practical framework for operationalizing local validation, monitoring and governance
Source: Front Radiol. 2026 Jul 16;6:1902217. doi: 10.3389/fradi.2026.1902217 (PMC13422437; doi:10.3389/fradi.2026.1902217)

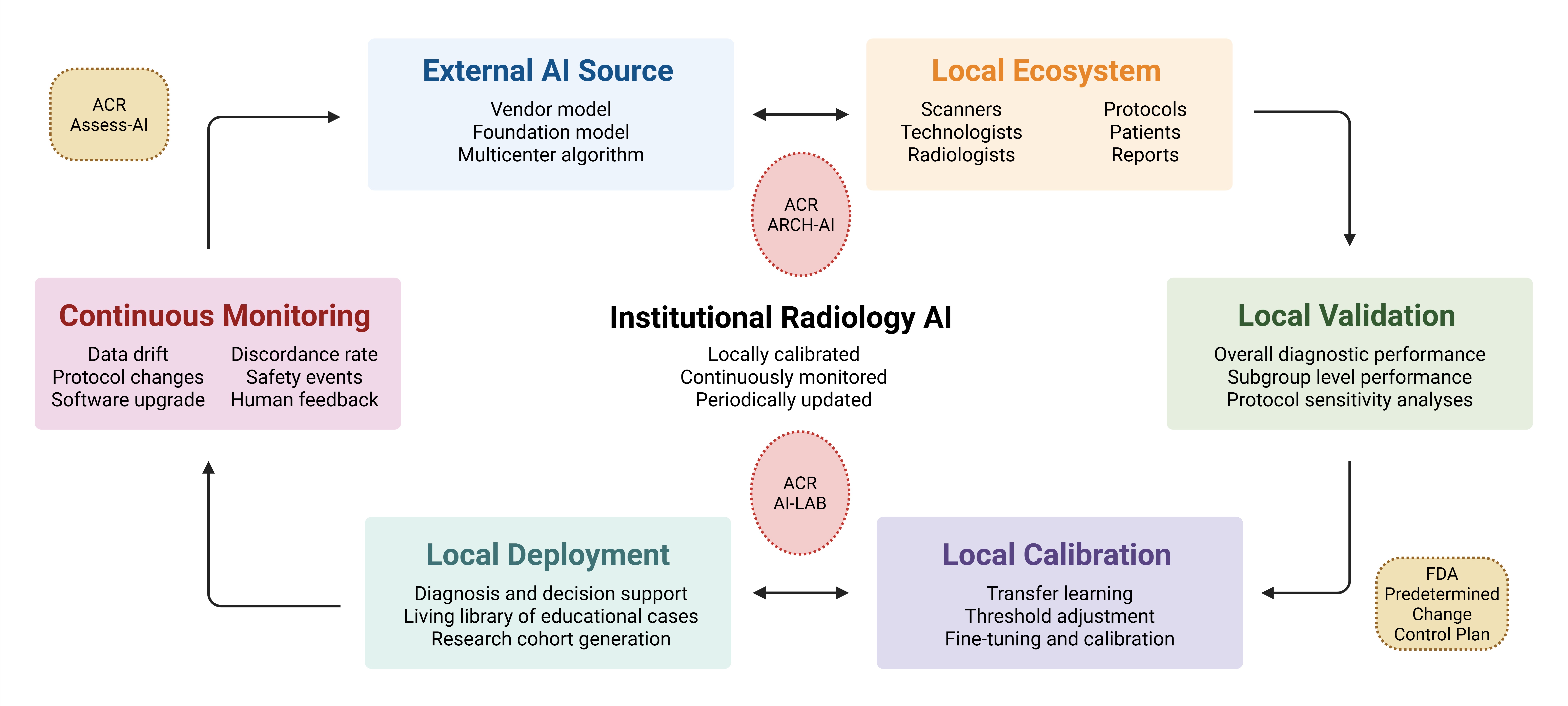

Supplement: Supplementary file 1 [file Image1.jpeg]
