# Supplementary material for: From plug-and-play to institution-calibrated radiology AI: a practical framework for operationalizing local validation, monitoring and governance
Source: Front Radiol. 2026 Jul 16;6:1902217. doi: 10.3389/fradi.2026.1902217 (PMC13422437; doi:10.3389/fradi.2026.1902217)
Supplement: Supplementary file 2 [file Supplementaryfile1.docx]

**Supplementary Material**


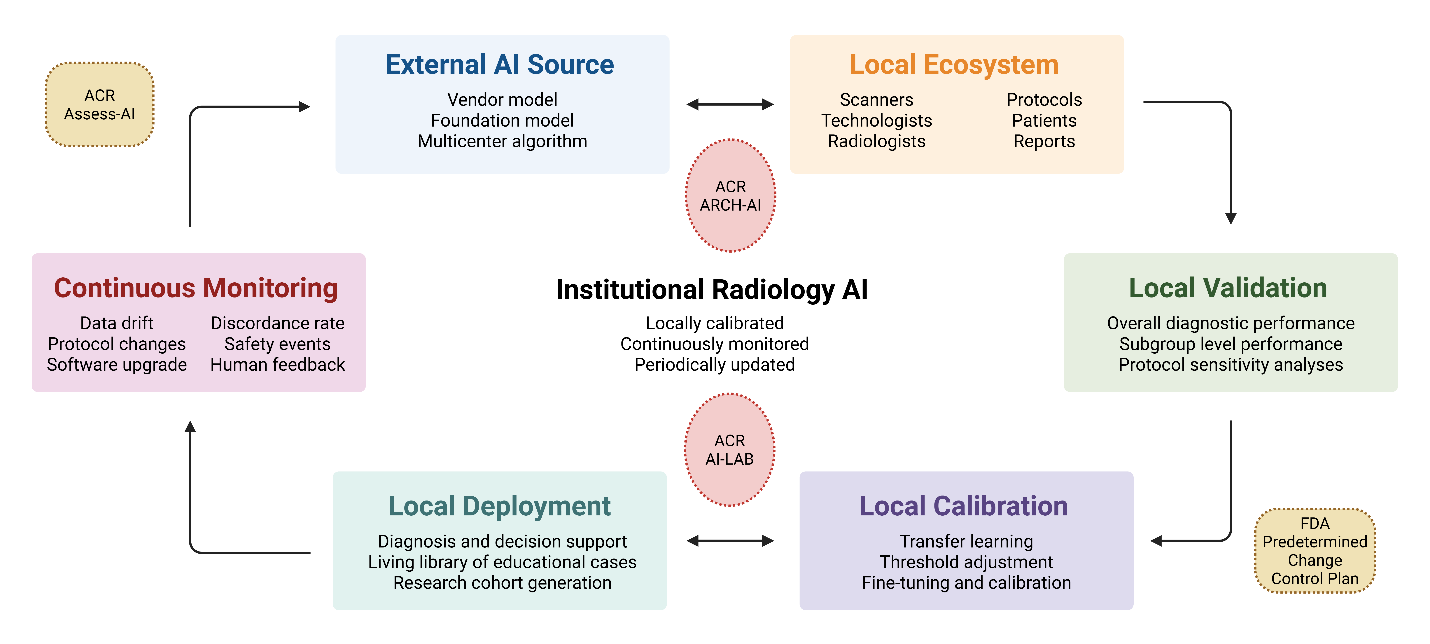


**Supplementary Figure 1: The institutional radiology AI performance profile: a framework for institution-calibrated AI deployment.** External/vendor/foundation models enter a local imaging ecosystem shaped by scanner hardware, acquisition protocols, technologist practices, patient populations, and reporting language. Local validation, adaptation when appropriate, deployment across diagnosis, research, and education, and continuous monitoring form a feedback loop. For example, within the United States, ACR AI-LAB, ARCH-AI, Assess-AI, and FDA Predetermined Change Control Plan guidance would provide complementary infrastructure for local evaluation, governance, monitoring, and regulatory guardrails. In other settings, WHO AI ethics and governance, NICE guidance, and ESR’s recommendations for effective AI implementation would play analogous roles. and Figure created using BioRender (https://BioRender.com/47s61ra).

**Supplementary Table 1: Published works and guidance regarding deployment of ML models in clinical environments vis-à-vis our proposal of an institutional radiology AI performance profile.**

| Prior work | Contributions of prior work | Similarities to our framework | Key differences |
| --- | --- | --- | --- |
| CyclOps | A software toolkit for cyclical development and operationalization of clinical ML models, including model development, evaluation, deployment, monitoring, subpopulation evaluation, drift detection, and model report cards. | Conceptual overlap in cyclical development, evaluation, monitoring, subpopulation performance, and model reporting. | CyclOps is primarily a technical software/API toolkit for developing and monitoring ML models across healthcare use cases. Our framework specifies what a radiology should summarize locally—by scanner, protocol, task, subgroup, and workflow—when those tools are specifically applied to radiology AI. |
| Resilience-aware MLOps | Extends MLOps for AI-based diagnostic systems by adding resilience to adversarial attacks, fault injection, drift, out-of-distribution data, uncertainty calibration, uncertainty monitoring, and graceful degradation. | Conceptual overlap regarding drift monitoring, uncertainty/calibration, robustness, and ongoing surveillance after deployment. | Resilience-aware MLOps addresses how to make AI diagnostic systems robust to disturbances; our framework identifies the institutional radiology domains where those disturbances become clinically visible and actionable from a radiologist’s perspective. |
| MLHOps | A broad healthcare MLOps framework covering reliable, efficient, usable, and ethical deployment of ML models, including data sources, pipeline engineering, deployment, monitoring, updating, bias, fairness, interpretability, and privacy. | Overlap in principle regarding lifecycle thinking, deployment, monitoring, data shift, fairness, ethics, and privacy. | MLHOps describes the general healthcare operational lifecycle; our radiology AI performance profile is a specialty-specific implementation artifact that operationalizes MLHOps inside radiology departments. |
| Five pillars of Clinical Deployment Environment | Proposes a Clinical Deployment Environment with five pillars: real-world development using live data, ML-Ops for health, responsible AI, implementation science, and continuous evaluation. It emphasizes infrastructure for development, deployment, and evaluation inside healthcare institutions. | Conceptual overlap regarding local deployment, continuous evaluation, stewardship, implementation science, and the need for infrastructure inside the institution. | The CDE describes the institution-level environment required for translational ML; our framework provides a radiology department-level dashboard/report card that could live within such a broader environment. |
| MedMLOps | A radiology-focused operational framework for medical ML systems, emphasizing availability, continuous monitoring, validation, retraining, privacy, data protection, ease of use, interoperability, safe retraining, and sustained reliability. | Closest to our framework in terms of addressing radiology AI deployment, monitoring, validation, retraining, privacy, safety, and reliability. | MedMLOps defines how radiology AI can be operationalized; our proposed radiology AI performance profile defines what should be measured and reported locally to make that operationalization clinically transparent and relevant for radiologists. |
| RSNA and MICCAI perspectives | Expert perspective on clinical, cultural, computational, and regulatory considerations for deploying AI in radiology, emphasizing trust, reproducibility, explainability, accountability, multidisciplinary collaboration, integration of imaging and clinical data, and incentivized clinical integration. | Overlap in trust, reproducibility, accountability, regulatory awareness, clinical integration, and multidisciplinary collaboration. | RSNA and MICCAI perspectives outline the cultural, computational, and regulatory conditions for AI adoption; our article translates these conditions into a local radiology performance-monitoring artifact. |
| Guidance from ESR, WHO, NICE, RANZCR, RSNA and ACR for purchasing, implementing, and monitoring AI tools | A practical radiology society statement addressing problems, ethical issues, safe product evaluation, clinical implementation, monitoring of stability and safety, and long-term integration of radiology AI. | Strong overlap in evaluation before adoption, safety monitoring, ethical/practical issues, and long-term integration. | The multisociety guidance tells departments that radiology AI must be evaluated, monitored and implemented meticulously; our paper proposes a concrete, structured, local performance profile for doing so in practice in a specific manner. |

Abbreviations: ACR, American College of Radiology; AI: artificial intelligence; ESR, European Society of Radiology; MICCAI, Medical Image Computing and Computer Assisted Intervention; ML, machine learning; NICE, National Institute for Clinical Excellence; RANZCR, Royal Australian and New Zealand College of Radiologists; RSNA, Radiologic Society of North America; WHO, World Health Organization.

**Supplementary Table 2: A prototype institutional radiology AI performance profile with suggested minimum elements for local validation, surveillance, and governance of imaging AI tools.**

| Profile component | Minimum elements | Stratification / cadence | Action triggered |
| --- | --- | --- | --- |
| Intended use and technical context | Task, model name and version, output type, scanner/vendor, protocol, care setting | By scanner, protocol, site, care setting; repeat after upgrades or protocol changes | Acceptance testing, metadata correction, or non-deployment if context is outside intended use |
| Pre-deployment performance | Sensitivity, specificity, PPV, NPV, AUROC, AUPRC, alert burden, radiologist concordance | Representative local test set; enrich rare but high-risk findings | Deploy, silent trial, local threshold review, or reject for local use |
| Reference standard | Expert adjudication, pathology, follow-up imaging, clinical outcomes, tumor board or M&M review, NLP-assisted follow-up | Focused review of discordant cases and safety events, at least quarterly where feasible | Update ground-truth labels, investigate false negatives, refine surveillance sample |
| Post-deployment workflow | Alert volume, override rate, report turnaround time, downstream testing, user feedback | Monthly or quarterly; triggered by workflow complaints or sudden alert changes | Workflow redesign, retraining of users, vendor escalation, or temporary pause |
| Equity and subgroup performance | Age, sex, race/ethnicity, insurance status, body habitus, comorbidity, rare conditions, care setting | Analyze when case numbers permit; use enriched review or registries for sparse groups | Bias mitigation plan, subgroup warning, additional validation, or restricted deployment |
| Governance and regulatory status | Owner, audit trail, version history, threshold changes, retraining status, vendor contact | Every version change, threshold change, or regulatory update | Determine local validation vs. permitted calibration vs. device modification requiring further review |

Abbreviations: AI: artificial intelligence; AUPRC, area under precision-recall curve; AUROC, area under receiver operating characteristic curve; ED, emergency department; M&M, morbidity and mortality; NLP, natural language processing; NPV, negative predictive value; PPV, positive predictive value.

**Supplementary Table 3: Consumers of the institutional radiology AI performance profile with examples of potential uses.**

| Consumer group | Required information furnished | Potential use |
| --- | --- | --- |
| Frontline radiologists | Whether the AI tool is reliable in their local workflow, where it fails, and when to distrust it | For example, this PE detection model has high PPV on ED CTA but sensitivity drops if contrast bolus timing is imperfect or streak artifacts from hardware degrade image quality. |
| Subspecialty section chiefs | Scanner/protocol-specific and service-line-specific performance | For instance, neuroradiology chief reviews whether an ICH triage tool performs differently on ED vs inpatient CT scans. |
| Radiology AI governance committee | Overall model inventory, validation status, drift, safety events, equity, and escalation triggers | Decide whether to continue, pause, recalibrate, or renegotiate a vendor tool. |
| Imaging informatics, PACS and IT teams | Integration performance, alert routing, uptime, interface failures, version changes | Identify whether alert delays are due to model performance or workflow integration. |
| Medical physicists | Scanner, acquisition protocol, reconstruction, and image-quality effects | Detect performance drop after scanner software upgrade or protocol change. |
| Quality and safety teams | Discordance, false negatives, false positives, near misses, and incident review | Incorporate AI failures into peer review, M&M, or QI dashboards. |
| Executive leadership | ROI, alert burden, throughput, downstream testing, maintenance cost, and clinical value | Decide whether to renew, expand, or discontinue an AI product. |
| Compliance, legal, and risk management | Documentation of validation, monitoring, change control, and human oversight | Demonstrate that AI was deployed with appropriate governance and surveillance. |
| Researchers and data scientists | Local PPV/recall of AI or NLP retrieval tools before using them for research cohorts | Determine whether AI-identified PE cases require manual adjudication before analysis. |
| Residency/fellowship educators | Locally validated case-retrieval pipelines and AI-discordant cases for teaching | Build teaching files from missed fractures, subtle pneumothorax, or protocol-specific pitfalls. |
| Vendors and developers | Site-specific failure modes and post-market performance feedback | Improve model robustness or provide better threshold/configuration options. |

Abbreviations: AI: artificial intelligence; CT, computed tomography; CTA, CT angiography; ED, emergency department; ICH, intracranial hemorrhage; IT, information technology; M&M, morbidity and mortality; NLP, natural language processing; PACS, picture archival and communication system; PE, pulmonary embolism; PPV, positive predictive value; QI, quality improvement; ROI, return on investment.

**Supplementary Table 4: Technical feasibility of different levels of local ML model validation, threshold adjustment, calibration and associated liability.**

| Activity | Technical feasibility | Liability and regulatory considerations |
| --- | --- | --- |
| Local validation without changing the model | Feasible | Lowest risk as the institution is evaluating local performance, not modifying the device in any way. |
| Monitoring categorical outputs after deployment | Feasible | Appropriate quality and safety activity; should be properly documented and governed with well-defined escalation criteria. |
| Changing workflow around alerts | Usually feasible | Generally safe but should be documented properly as it may affect radiologist and clinician behavior. |
| Changing a vendor-provided configurable threshold | Feasible only if the vendor provides this option | Acceptable if done within labeling, contract, vendor guidance, and regulatory constraints. |
| Post-hoc recalibration using continuous scores | Feasible only if probability scores are available | Higher than usual risk; needs regulatory/legal review, validation, documentation, and clarity on whether this creates a modified device or local decision-support layer. |
| Fine-tuning/retraining a commercial model | Usually not feasible | Highest risk as it may constitute a device modification and shift liability toward the modifying party, unless performed under vendor/regulatory pathway. |
| Accessing or altering model weights | Usually not feasible | Not recommended for routine institutional deployment; requires vendor involvement and likely regulatory review. |

Abbreviations: ML, machine learning.

**Supplementary Table 5: Illustrative resource and cost-allocation structure* for maintaining an institutional radiology AI performance profile.**

| Resource domain | Core responsibilities | Minimal viable approach | Illustrative commitment for average-sized hospital | Potential cost categories | Potential value offsets |
| --- | --- | --- | --- | --- | --- |
| Radiologist clinical champion | Define intended use, review discordant cases, interpret clinical significance of performance metrics, lead escalation decisions. | Assign one radiologist with protected quality/safety or informatics time. | 0.1–0.2 FTE radiologist champion; higher during initial validation or major implementation. | Protected physician time, meeting time, peer-review effort. | Improved clinical trust, earlier recognition of unsafe performance, reduced inappropriate reliance on AI outputs. |
| Imaging informatics / data analyst support | Extract local cases, link AI outputs to PACS/RIS/EHR data, generate dashboards, track alert volume and performance drift. | Use existing PACS/RIS analytics or quality dashboard infrastructure when available. | 0.2–0.5 FTE informaticist or analyst; may increase for multi-site or multi-model deployments. | Analyst/informaticist salary support, dashboard software, data pipeline maintenance. | Reduced manual audit burden, improved vendor accountability, more efficient performance surveillance. |
| Medical physicist / modality lead input | Assess scanner, protocol, reconstruction, field strength, dose, and image-quality factors that may affect model performance. | Include physicist review after scanner upgrades, protocol changes, or unexpected performance shifts. | Ad hoc review plus quarterly or semiannual participation in AI quality meetings. | Physicist time, protocol review, image-quality audits. | Earlier detection of hardware/protocol-related performance degradation; fewer preventable technical failure modes. |
| Quality and safety infrastructure | Integrate AI monitoring with peer review, discrepancy review, morbidity/mortality conferences, safety-event reporting, and QI dashboards. | Embed AI review into existing radiology QI workflows rather than creating a separate parallel process. | Existing QI committee review plus focused AI agenda items monthly or quarterly. | QI personnel time, committee time, documentation and audit support. | Reduced duplication, stronger audit trail, improved response to near misses and false-negative events. |
| Ground truth adjudication | Establish reference standards for validation and surveillance, including expert review of discordant cases and enriched case samples. | Manual review of a targeted sample of positive, negative, and discordant cases. | 20–50 cases per model per quarter for surveillance; larger samples for initial validation or high-risk tools. | Radiologist adjudication time, chart review time, research/QI coordinator support. | More reliable monitoring, clearer understanding of false-positive and false-negative patterns. |
| IT / cybersecurity / vendor-management support | Maintain integration, uptime, interface monitoring, version tracking, cybersecurity review, and vendor communication. | Add AI tools to existing IT change-control and vendor-management processes. | Ad hoc support during implementation; periodic review after software updates or integration failures. | Interface maintenance, cybersecurity review, vendor contract management. | Reduced downtime, faster troubleshooting, improved contract negotiation and service-level accountability. |
| Governance committee | Review validation results, approve deployment, monitor drift, define escalation triggers, and decide whether to continue, pause, modify, or retire AI tools. | Use an existing AI, informatics, quality, or radiology operations committee. | 30–60-minute meeting monthly during implementation; quarterly once stable. | Administrative support, committee time, documentation. | Improved accountability, clearer liability boundaries, better procurement and renewal decisions. |
| Equity and population-health support | Evaluate subgroup performance, demographic missingness, care access, follow-up completion, and potential differential impact. | Include equity review for high-impact models or when subgroup signals emerge. | Periodic consultation with data governance, DEI, population health, or social determinants teams. | Data access support, privacy review, population-health analyst time. | Earlier detection of differential performance or access-related harm; improved institutional trust. |
| Legal, compliance, and risk management | Clarify whether validation, workflow configuration, threshold adjustment, or retraining creates regulatory or liability implications. | Review only high-risk changes, vendor-supported threshold changes, or deviations from product labeling. | Ad hoc review before major implementation, model modification, or workflow change affecting clinical use. | Legal/compliance review time, documentation, risk-management consultation. | Reduced medicolegal exposure, clearer documentation of human oversight and change control. |
| Administrative and financial oversight | Track direct costs, indirect costs, maintenance fees, productivity effects, and renewal decisions. | Include AI performance and resource use in value-analysis or technology-review committees | Annual review of cost, utilization, alert burden, performance, and clinical value. | Vendor fees, software maintenance, personnel support, opportunity cost. | Better return-on-investment assessment, discontinuation of low-value tools, reduced alert fatigue, improved procurement decisions. |

* Estimates are illustrative only and should be scaled to actual institutional size, number of AI tools, clinical risk, imaging volume, and available infrastructure. Initial deployment requires the greatest effort; ongoing surveillance may be less resource-intensive if integrated into existing radiology quality, informatics, and peer-review workflows.

Abbreviations: AI, artificial intelligence; DEI, diversity, equity, and inclusion; EHR, electronic health record; FTE, full-time equivalent; IT, information technology; PACS, picture archival and communication system; QI, quality improvement; RIS, radiology information system.

**Supplementary Table 6: A sample practical checklist for equity-focused subgroup evaluation of institutional radiology AI performance.**

| Domain | Assessment | Metrics | Practical considerations |
| --- | --- | --- | --- |
| Age | Stratify model performance by clinically meaningful age groups, such as pediatric, adult, and older adult populations, or by age bands relevant to the imaging task. | Sensitivity, specificity, PPV, NPV, false-negative rate, false-positive rate, alert burden. | Avoid overinterpreting unstable estimates in small age strata. Use wider age bands or pooled monitoring periods when needed. |
| Sex | Evaluate performance separately by sex when biologically or clinically relevant to the imaging task. | Diagnostic performance metrics, alert rate, radiologist override rate, discordance rate. | Consider whether sex-specific differences may reflect disease prevalence, anatomy, acquisition technique, or referral patterns. |
| Race and ethnicity | Examine whether model performance differs across locally recorded race and ethnicity categories. | Sensitivity, specificity, PPV, NPV, false-negative rate, false-positive rate, missing-data rate. | Race and ethnicity are imperfect social variables and may be incompletely documented. Collaborate with institutional DEI, data governance, or population health teams to ensure appropriate interpretation and privacy protection. |
| Body habitus | Assess performance across BMI categories or other proxies for body habitus when image quality may affect AI output. | Image-quality failure rate, nondiagnostic rate, false-negative rate, false-positive rate, alert rate, confidence/probability distribution if available. | Body habitus may interact with modality, scanner protocol, dose, reconstruction method, and image noise. Interpret findings with physicist and modality-lead input. |
| Care setting | Compare performance across emergency department, inpatient, outpatient, screening, and procedural settings. | Alert rate, turnaround time, PPV, false-positive burden, radiologist override rate, downstream testing. | Differences may reflect disease prevalence and workflow rather than model bias alone. Stratification by indication may be necessary. |
| Imaging access and affordability | Assess whether model performance, alert burden, follow-up completion, and downstream outcomes differ by access-related variables such as insurance status, self-pay status, referral source, distance from imaging site, outpatient versus emergency imaging pathway, or availability of prior imaging. | Follow-up completion rate, time to recommended imaging, missed follow-up rate, downstream testing, PPV, false-negative review findings, alert-to-action time, care delays after AI flagging. | Access variables may reflect structural barriers rather than model bias alone. Interpret results with population health, care coordination, social work, and institutional equity teams. Avoid using access-related variables to restrict AI use; instead, use them to identify where AI workflows may worsen or mitigate disparities. |
| Socioeconomic status | Evaluate performance and downstream impact by individual-level income when available, or by area-level proxies such as ZIP-code–linked median household income, deprivation index, insurance type, or neighborhood socioeconomic measures. | Sensitivity, specificity, PPV, NPV, alert rate, follow-up completion, time to diagnosis, time to intervention, downstream utilization, missed-care opportunities. | Income is often unavailable or estimated indirectly. Area-level income is an ecological proxy and should not be interpreted as individual income. Small numbers and missingness are common. Use analyses primarily to detect inequities in access, follow-up, and clinical action after AI output rather than to infer biologic differences in model performance. |
| Uncommon conditions and underrepresented disease patterns | Examine performance for rare cancers, uncommon complications, atypical presentations, and low-prevalence findings where training data may have been sparse. | Case-level review of false negatives and false positives, detection rate, PPV, time to diagnosis, expert adjudication outcomes. | Formal subgroup statistics may be impossible. Use qualitative error review, enriched case audits, or targeted expert adjudication rather than relying only on aggregate metrics. |
| Intersectional subgroups | Where feasible, examine clinically meaningful intersections, such as older adults with high BMI, women with specific disease presentations, or underrepresented groups imaged on lower-volume scanners. | Descriptive performance estimates, discordance review, false-negative patterns, alert burden. | Intersectional analyses may be underpowered. Use them for signal detection and QI prioritization rather than definitive hypothesis testing. |
| Missing or unreliable demographic data | Quantify missingness and assess whether missing demographic data are associated with care setting, site, or imaging indication. | Missing-data rate, completeness by site or workflow, sensitivity analyses with and without missing categories. | Missing demographic data should not be ignored. High missingness may limit equity conclusions and should be transparently reported. |
| Governance and response plan | Define who reviews subgroup performance and what actions follow a concerning signal. | Review by AI governance committee, radiology quality/safety team, DEI or population health representatives, informatics, physicists, and vendor when appropriate. | Predetermined escalation criteria: additional case review, workflow modification, protocol review, vendor notification, temporary restriction of use, or revalidation. |

Abbreviations: AI, artificial intelligence; BMI, body mass index; DEI, diversity, equity, and inclusion; M&M, morbidity and mortality; NPV, negative predictive value; PPV, positive predictive value; QI, quality improvement.
